# Supplementary figures and images for: Alternation emerges as a multi-modal strategy for turbulent odor navigation
Source: eLife. 2022 Aug 23;11:e76989. doi: 10.7554/eLife.76989 (PMC9489216; doi:10.7554/eLife.76989)

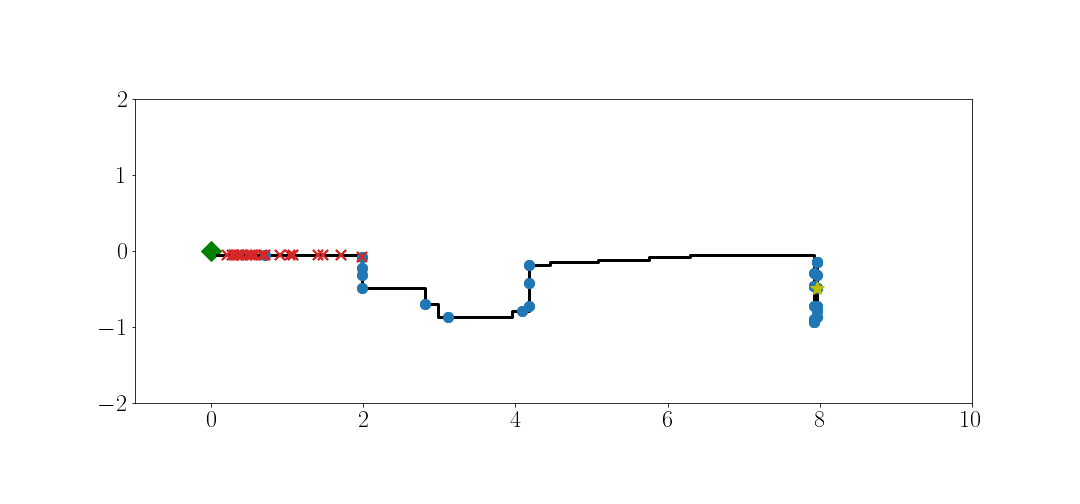

Supplement: Supplementary file 1. [file elife-76989-supp1.zip › data_availability/fig3/Traj_3a.png]

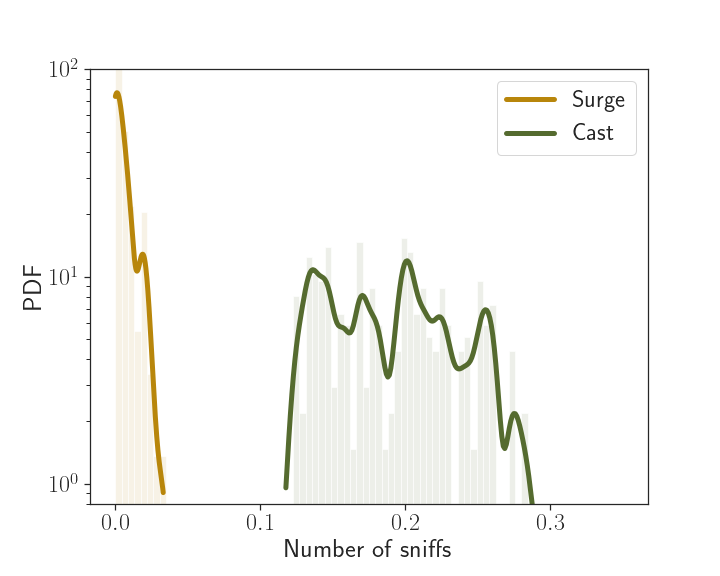

Supplement: Supplementary file 1. [file elife-76989-supp1.zip › data_availability/fig4/Figure_4c.png]

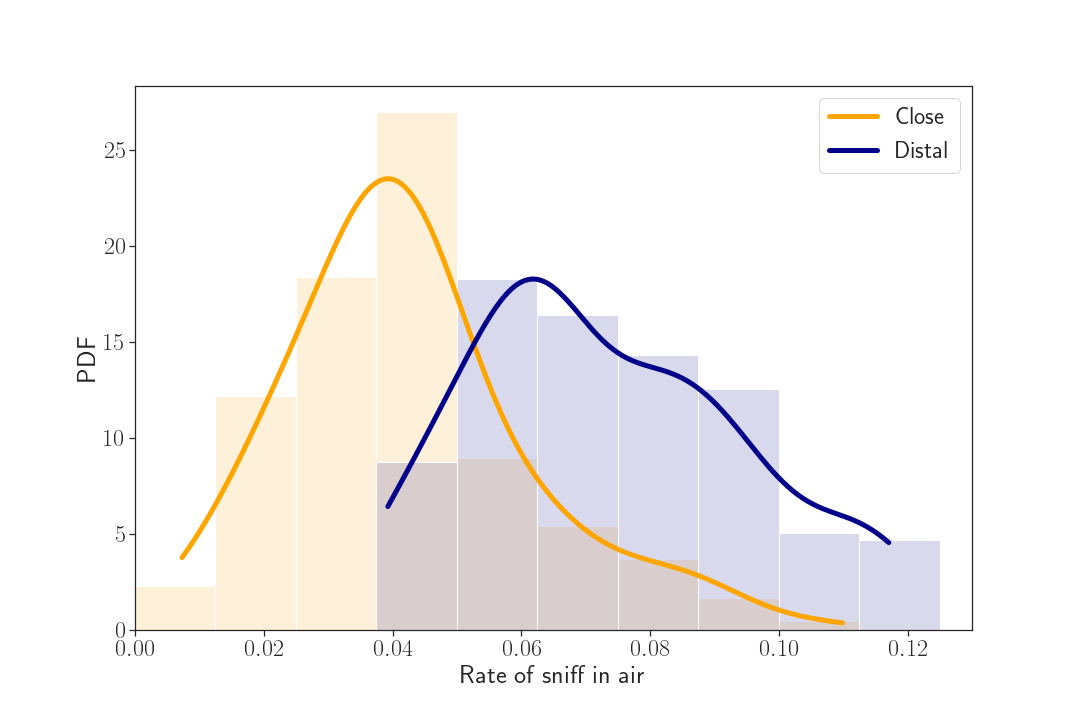

Supplement: Supplementary file 1. [file elife-76989-supp1.zip › data_availability/fig4/figure_4A.png]
