# Supplementary material for: Alternation emerges as a multi-modal strategy for turbulent odor navigation
Source: eLife. 2022 Aug 23;11:e76989. doi: 10.7554/eLife.76989 (PMC9489216; doi:10.7554/eLife.76989)
Supplement: Transparent reporting form [file elife-76989-transrepform1.docx]

***eLife’s* transparent reporting form**

We encourage authors to provide detailed information *within their submission* to facilitate the interpretation and replication of experiments. Authors can upload supporting documentation to indicate the use of appropriate reporting guidelines for health-related research (see [EQUATOR Network](http://www.equator-network.org/%20)), life science research (see the [BioSharing Information Resource](https://biosharing.org/)), or the [ARRIVE guidelines](http://www.plosbiology.org/article/info:doi/10.1371/journal.pbio.1000412) for reporting work involving animal research. Where applicable, authors should refer to any relevant reporting standards documents in this form.

If you have any questions, please consult our Journal Policies and/or contact us: [editorial@elifesciences.org](mailto:editorial@elifesciences.org).

**Sample-size estimation**

- You should state whether an appropriate sample size was computed when the study was being designed
- You should state the statistical method of sample size computation and any required assumptions
- If no explicit power analysis was used, you should describe how you decided what sample (replicate) size (number) to use

Please outline where this information can be found within the submission (e.g., sections or figure legends), or explain why this information doesn’t apply to your submission:

For convergence of the POMDP, we set the number of replicates (number of epochs for training) so that performance of the searcher would plateau (see Supplementary Figure 1). The number of trajectories that we tested the algorithm with is described in the Materials and Methods -- Parameters for POMDP used in the main text.

The manuscript contains no experimental data.

**Replicates**

- You should report how often each experiment was performed
- You should include a definition of biological versus technical replication
- The data obtained should be provided and sufficient information should be provided to indicate the number of independent biological and/or technical replicates
- If you encountered any outliers, you should describe how these were handled
- Criteria for exclusion/inclusion of data should be clearly stated
- High-throughput sequence data should be uploaded before submission, with a private link for reviewers provided (these are available from both GEO and ArrayExpress)

Please outline where this information can be found within the submission (e.g., sections or figure legends), or explain why this information doesn’t apply to your submission:

n/a, No experiments were performed

The number of replicates of the simulations is specified in the Materials and Methods -- Parameters for POMDP used in the main text.

**Statistical reporting**

- Statistical analysis methods should be described and justified
- Raw data should be presented in figures whenever informative to do so (typically when N per group is less than 10)
- For each experiment, you should identify the statistical tests used, exact values of N, definitions of center, methods of multiple test correction, and dispersion and precision measures (e.g., mean, median, SD, SEM, confidence intervals; and, for the major substantive results, a measure of effect size (e.g., Pearson's r, Cohen's d)
- Report exact p-values wherever possible alongside the summary statistics and 95% confidence intervals. These should be reported for all key questions and not only when the p-value is less than 0.05.

Please outline where this information can be found within the submission (e.g., sections or figure legends), or explain why this information doesn’t apply to your submission:

In Figure 4 we analyse the sniff rate, showing the probability density functions constructed by binning the sniff rates events (the size of the bins is directly visible in the figure, and the statistics is performed over the ensemble described in the Materials and Methods -- Parameters for POMDP used in the main text). The statistics showed in Figure 4b are described in the caption.

The results for POMDP are obtained using the parameters and statistics described in the Materials and Methods -- Parameters for POMDP used in the main text.

(For large datasets, or papers with a very large number of statistical tests, you may upload a single table file with tests, Ns, etc., with reference to sections in the manuscript.)

**Group allocation**

- Indicate how samples were allocated into experimental groups (in the case of clinical studies, please specify allocation to treatment method); if randomization was used, please also state if restricted randomization was applied
- Indicate if masking was used during group allocation, data collection and/or data analysis

Please outline where this information can be found within the submission (e.g., sections or figure legends), or explain why this information doesn’t apply to your submission:

n/a, no experiments were performed

**Additional data files (“source data”)**

- We encourage you to upload relevant additional data files, such as numerical data that are represented as a graph in a figure, or as a summary table
- Where provided, these should be in the most useful format, and they can be uploaded as “Source data” files linked to a main figure or table
- Include model definition files including the full list of parameters used
- Include code used for data analysis (e.g., R, MatLab)
- Avoid stating that data files are “available upon request”

Please indicate the figures or tables for which source data files have been provided:

We provide all dataset used for figures 2c,3,4,5,6g, where statistical analysis of the POMDP is performed.

Additionally, we include the scripts computing the posterior based on the trajectory and the likelihood and produce Figure 5, as well as the scripts that produce the pdfs in Figure 4a and 4c; the visualization of the POMDP behaviour in Figure 3; as well as the statistics in figure 6g. These should help to easily reproduce the figures and manipulate the data.

The data are shared in the form of a zipped folder; the format is described within the data_availability Folder in a readme file.
